# Health, safety training, and exposure reduction practices among U. S. fire investigators: a cross-sectional study

**DOI:** 10.3389/fpubh.2026.1806507

**Published:** 2026-05-14

**Authors:** Alberto J. Caban-Martinez, Anton Stremousov, Madeleine M. Sayre, Tulay Koru-Sengul, Sylvia Daunert, Christopher Weidlich, Kristopher J. Paultre, Jeffrey L. Pauley, Natasha Schaefer Solle

**Affiliations:** 1 Department of Public Health Sciences, Leonard M. Miller School of Medicine, Miami, FL, United States; 2 Leonard M. Miller School of Medicine, Sylvester Comprehensive Cancer Center, Miami, FL, United States; 3 Department of Orthopaedics, Leonard M. Miller School of Medicine, Miami, FL, United States; 4 Department of Biochemistry and Molecular Biology, Leonard M. Miller School of Medicine, Miami, FL, United States; 5 School of Nursing and Health Studies, Coral Gables, FL, United States; 6 Pacific Pointe Consulting, Summerfield, NC, United States; 7 Department of Medicine, Leonard M. Miller School of Medicine, Miami, FL, United States

**Keywords:** fire investigators, fire scene investigation, occupational exposure, occupational health, personal protective equipment, photovoice

## Abstract

**Background:**

Fire investigators conduct post-fire scene work with potential exposure to combustion byproducts, damaged structures, and psychosocial stressors, yet data on exposure reduction practices and health characteristics remain limited. The objective of this analysis is to characterize training, protective practices, self-reported exposures, and health indicators among U. S. fire investigators.

**Methods:**

As part of a national photovoice study, fire investigators completed a confidential pre-interview survey between April and November 2025. Survey items assessed demographics, occupational history, use of personal protective equipment and respiratory protection, exposures, safety training, decontamination practices, and self-reported health conditions and behaviors.

**Results:**

Fifty-six investigators from 24 states participated (mean 12.6 years of experience); 71.4% were public investigators and 29.6% had a second job. Personal protective equipment use during investigations was reported as often or always by 92.9%, while 60.7% reported often or always using respiratory protection. Exposures reported as often or always included soot (89.3%), burned debris (73.2%), smoke (67.9%), and hazardous substances (60.7%). Psychosocial stressors occurred often or always for 28.6%. Preliminary exposure reduction training was reported by 12.5, and 56.4% indicated their agency lacked investigator-specific decontamination practices. Self-rated health was reported as very good or good by 83.9%. Self-reported conditions included cardiovascular disease (21.4%), musculoskeletal disorders (17.9%), mental health conditions (12.5%), and cancer (5.4%).

**Conclusion:**

U. S. fire investigators report frequent post-fire exposures and variability in respiratory protection use, exposure reduction training, and investigator-specific decontamination policies. This pilot study data can inform occupational health guidance and future research on exposure assessment, health monitoring, and control strategies.

## 1 Introduction

Fire investigators are a specialized public safety workforce responsible for determining the origin and cause of a fire, documenting scene conditions, and supporting prevention activities and legal proceedings (1, 2). Investigative tasks often occur during overhaul or in the days following suppression, when debris disturbance, limited ventilation, and damaged structures can create ongoing chemical and physical hazards (3). Controlled residential fire studies have shown that airborne contamination during post-fire investigation can include substantial particulate matter and reactive gases such as formaldehyde, indicating that potentially hazardous exposures may persist beyond active firefighting (4). Field-based occupational evaluations likewise document exposures to irritants and combustion byproducts during investigative work, including measurable formaldehyde at fire scenes (5). Together, these findings have increased interest in defining investigator-specific exposure pathways and safety controls that match the realities of post-fire scene work, including contaminants such as polycyclic aromatic hydrocarbons (PAHs) measured with passive sampling approaches (6).

Beyond documenting the presence of these hazards, it is important to consider their potential health relevance for fire investigators. Post-fire contaminants such as fine particulate matter, aldehydes, and other combustion byproducts may contribute to acute respiratory irritation, cough, wheeze, reduced lung function, and pulmonary inflammation (4, 7). At the same time, while repeated exposure to irritants such as formaldehyde may also be associated with asthma-like symptoms and dermatitis (8). Formaldehyde is additionally classified as a human carcinogen, and PAHs generated during incomplete combustion are well-established toxicants of occupational concern, with several compounds and related exposures linked to elevated cancer risk, particularly for lung and skin outcomes (8). In parallel, the organizational demands and potentially traumatic nature of fire investigation work may affect mental health and well-being, as fire-service psychosocial stressors have been associated with depression, sleep disturbance, burnout, alcohol misuse, and post-traumatic stress symptoms (9).

In contrast to the expanding literature on firefighter occupational health (9–12), the evidence base for fire investigators remains comparatively sparse, and investigator-focused surveillance has lagged behind. This gap is notable given the scale of the broader U. S. fire service workforce, which has been estimated at roughly 1.1 million active firefighters by the National Fire Protection Association (13). By comparison, fire investigators constitute a much smaller segment of this workforce, with the U. S. Bureau of Labor Statistics reporting national employment of approximately 14,050 for fire inspectors and investigators (14). The relatively small workforce size, heterogeneous roles (public sector, law enforcement, private sector), and decentralized employment structures can limit study power and contribute to fragmented evidence on exposures, health outcomes, and protective practices. As a result, we still lack a comprehensive understanding of the overall health and safety of the fire investigator workforce, including how work organization, work environment and control measures shape their cumulative risk for injuries and diseases.

To help address this knowledge gap, we conducted a qualitative study with 56 fire investigators recruited from across the United States. As part of the study interview procedures, we assessed participant characteristics and work patterns alongside self-reported scene hazards, use of personal and respiratory protective equipment, training and decontamination practices, and selected health indicators that may be relevant to occupational exposures and work-related stressors encountered during fire investigation. In the present analysis, we summarize survey data collected from these fire investigators regarding their socio-demographics, employment characteristics, safety practices and exposures, and health conditions and behaviors. By characterizing this understudied subgroup within the fire service, this study aims to inform future analytic epidemiology, intervention development, and surveillance efforts tailored to fire investigator work. Although these descriptive data cannot establish causation, characterizing potential overlap between reported occupational hazards and reported health conditions may help generate hypotheses for future analytic epidemiology and targeted prevention strategies.

## 2 Materials and methods

### 2.1 Study design and study sample

Pre-interview survey data collected between April to November 2025, as part of a qualitative study on the U. S. fire investigator workforce evaluating occupational health and safety were analyzed. Potential fire investigator participants were approached by the study team to explain the study purpose and study procedures. Interested fire investigators initially completed verbal consent prior to the start of any study procedures. Following consent, participants were asked to complete a pre-interview survey comprised of 48-items via an online, secure, and confidential REDCap platform (15, 16). Once the survey was complete, participants were asked to upload five digital photos that represent health promotions scenes at their fire agency, and five photos that represent health risks in their work environment. Participants were subsequently interviewed via Zoom using a photo-voice technique to documents perceptions, experiences and perspectives of occupational health and safety concerns. This study was approved by the Institutional Review Board (IRB) of the University (IRB #20250092).

### 2.2 Participant recruitment

Partnerships with the International Association of Arson Investigators, National Association of Fire Investigators, National Fire Protection Association, North American Fire Training Directors, Fire Department Safety Officers Association, and members of the study advisory board comprised of U. S. fire investigators supported not only the design of the study, but the recruitment of fire investigators across the United States. Study recruitment flyers were distributed and posted through professional fire investigator organizations, the advisory board membership and through fire department social media channels.

### 2.3 Study measures

#### 2.3.1 Pre-interview survey instrument

The survey was organized into 4 sections assessing demographic information (10-items), occupational history and job exposures (30-items), general health status, and mental and behavioral health status (8-items). Most question items where adapted from the National Health Interview Survey questionnaire and from prior firefighter health studies (17, 18). The questionnaire was reviewed, edited and approved by the study’s fire investigator advisory committee prior to being fielded with study participants.

#### 2.3.2 Demographic information measures

Question items assessing participant demographics captured information on the fire investigators chronological age, sex, race, ethnicity, highest level of educational attainment, marital status, self-reported height and weight, household composition including whether they had children, and total annual household income. These variables were collected to describe the study sample and were not intended as direct measures of take-home exposure risk in the present analysis.

#### 2.3.3 Occupational history/exposure measures

Question items assessing occupational history and work characteristics captured information on fire investigation training and certification status, including national and state-level fire investigation credentials. Participants reported their current status as an active fire investigator, years of experience in fire investigation, and average frequency of on-scene fire investigations per month. Participants also reported their average weekly time spent at fire investigation scenes using categorical response options. Additional items assessed prior to, concurrent with, and after employment history, including previous or current work as a firefighter or fire inspector, type of fire investigator role (public, private, or both), presence of secondary employment outside the fire service, and primary work setting. This measure was included to help characterize potential additional occupational demands and exposures outside fire investigation duties that could contribute to cumulative health and safety risk.

#### 2.3.4 Safety practices, training, and occupational exposure measures

Safety practices and occupational exposure measures assessed use of personal protective equipment (PPE) and personal protective respiratory equipment (PPRE) during fire investigations, including frequency of use and specific types worn. Participants reported frequency of exposure to common fire investigation hazards, including smoke, soot, hazardous substances such as gases and chemicals, burned or collapsed fire debris, psychosocial stressors, and bloodborne pathogens. Items also captured whether participants had received training related to preliminary exposure reduction or decontamination practices. Additional questions assessed PPE cleaning behaviors, methods of PPE cleaning, frequency of post-investigation decontamination, and whether formal decontamination policies specific to fire investigators were present within their department or agency.

#### 2.3.5 Health status and health behavior measures

Health-related measures captured self-rated overall health status and self-reported history of diagnosed health conditions, including cardiovascular, respiratory, metabolic, musculoskeletal, mental health, and cancer. Participants also reported family history of cancer and cardiovascular disease. Health behavior survey items assessed lifetime tobacco use, frequency of alcohol consumption, access to mental health resources through their department or agency, and history of seeking professional support for work-related stress or trauma.

### 2.4 Data analysis

Pre-interview survey data were analyzed using descriptive statistics to characterize participant survey responses and were carefully reviewed for completeness and internal consistency prior to analysis. Categorical variables were summarized using frequencies and percentages. These included demographic characteristics, employment history, certification status, use of personal protective equipment and respiratory protection, reported exposure frequencies, training experiences, and health conditions and behaviors. Continuous variables were summarized using measures of central tendency and dispersion. Specifically, years of tenure as a fire investigator were summarized using means and standard deviations, with minimum and maximum values reported to describe the range of experience. All analyses were conducted using IBM SPSS Statistics, version 31 (19).

## 3 Results

### 3.1 Sociodemographic characteristics

Of the 104 individuals who expressed interest in the study, 48 (46.2%) were excluded and 56 (53.8%) were included in the data analysis (Table 1). Among those excluded, 10 (20.8%) were either located outside of the United States or were not active fire investigators, and 38 (79.2%) expressed interest but did not enroll by completing the informed consent and/or the survey. Age groups were distributed across 28 to 39 years (26.4%), 40 to 49 years (35.8%), 50 to 59 years (34.0%), and 60 years or older (3.8%). Participants reported male sex (89.3%) and female sex (10.7%). Race was reported as White (96.4%), Black/African American (1.8%), and other (1.8%); ethnicity was reported as Hispanic/Latino (5.5%) and non-Hispanic (94.5%). Highest educational attainment included fire investigators who completed an associate degree (30.4%), bachelor’s degree (41.1%), and master’s degree (19.6%). BMI category was reported as healthy weight (5.5%), overweight (45.5%), and obese (49.1%). Annual household income was reported as $200,000 or more (37.0%), $150,000 to $199,999 (27.8%), $100,000 to $149,999 (27.8%), and $75,000 to $99,999 (7.4%). Most participants reported being married (92.9%) and having children (85.5%). Fire investigators enrolled in this study were from Arizona, California, Colorado, Connecticut, Florida, Indiana, Maryland, Maine, Minnesota, Missouri, North Carolina, New Hampshire, New Jersey, New Mexico, New York, Oregon, Pennsylvania, Rhode Island, South Dakota, Tennessee, Texas, Virginia, Washington, and West Virginia.

**Table 1:** Sociodemographic of participants in the fire investigators’ lived awareness of risk and exposure – a photovoice study on safety and health (*n* = 56).

Characteristics	*n* (%)^†^
28–39 years old	14 (26.4)
40–49 years old	19 (35.8)
50–59 years old	18 (34.0)
60 and older	2 (3.8)
Sex	
Male	50 (89.3)
Female	6 (10.7)
Race	
White	53 (96.4)
Black/African American	1 (1.8)
Other	1 (1.8)
Ethnicity	
Hispanic/Latino	3 (5.5)
Non-Hispanic	52 (94.5)
Educational attainment	
High School Diploma/GED	3 (5.4)
Associates degree	17 (30.4)
Bachelor’s degree	23 (41.1)
Master’s degree	11 (19.6)
Marital status	
Married	52 (92.9)
Divorced	2 (3.6)
Never Married	1 (1.8)
A member of an unmarried couple	1 (1.8)
Body mass index category	
Healthy weight (18.5 to 24.9 kg/m2)	3 (5.5)
Overweight (25.0 to 29.9 kg/m2)	25 (45.5)
Obese (30 kg/m2 or more)	27 (49.1)
Household income	
$75,000–$99,999	4 (7.4)
$100,000–$149,999	15 (27.8)
$150,000–$199,999	15 (27.8)
$200,000 or more	20 (37.0)
Has children	
No	8 (14.5)
Yes	47 (85.5)

^†^Differences in sub-total sample due to item non-response or missing.

### 3.2 Work and employment characteristics

Participants reported a mean tenure as a fire investigator of 12.6 years (SD 9.7; range 1 to 45) (Table 2). Monthly investigation frequency was reported as greater than 4 investigations (45.5%), 1 to 2 investigations (38.2%), and 3 to 4 investigations (16.4%). Prior work as a firefighter was reported by 90.9%, and prior work as a fire inspector was reported by 53.6%. Participants most frequently reported working as public investigators only (71.4%), followed by private investigators only (12.5%) and both public and private investigators (14.5%). A second job outside the fire service was reported by 29.6%. Current work setting was reported as urban (44.6%), suburban (35.7%), other (17.9%), and rural (1.8%). Time at fire scene investigations per week was reported as less than 10 h (66.1%), 10 to 19 h (25.0%), and 20 to 29 h (8.9%). Certifications reported included Certified Fire Investigator (CFI) through IAAI (36.4%), state certified fire investigator (36.4%), Certified Fire and Explosion Investigator (CFEI) through NAFI (12.7%), and other certifications.

**Table 2:** Work and employment characteristics of participants in the fire investigators’ lived awareness of risk and exposure – a photovoice study on safety and health (*n* = 56).

Characteristics	*n* (%)^†^
Fire certification held^‡^	
Certified Fire Investigator (CFI) (IAAI)	20 (36.4)
Certified Fire Investigation Instructor (CFII)	1(1.8)
Certified Fire and Explosion Investigator (CFEI) - NAFI	7(12.7)
State Certified Fire Investigator (if yes, specify state)	20(36.4)
Other	7(12.7)
Tenure as fire investigator	Mean ± Standard Deviation (SD)
Years ± SD	12.6 ± 9.7; min = 1 & max = 45
Average fire scene investigations	*n* (%)
1–2 Investigations per month	21 (38.2)
3–4 Investigations per month	9 (16.4)
Greater than 4 per month	25 (45.5)
Ever work as a firefighter	
No	5 (9.1)
Yes	50 (90.9)
Ever worked as fire inspector	
No	26 (46.4)
Yes	30 (53.6)
Type of fire investigator	
Public investigator only	40 (71.4)
Private investigator only	7 (12.5)
Both public and private investigator	8 (14.5)
Has second job outside fire service	
No	38 (70.4)
Yes	16 (29.6)
Current work setting	
Urban	25 (44.6)
Suburban	20 (35.7)
Rural	1 (1.8)
Other	10 (17.9)
Time since last fire investigation	
Same day or ≤ 48 h	14 (25.0)
3–7 days	6 (10.7)
8–14 days	12 (21.4)
15–30 days	9 (16.1)
> 30 days	11 (19.6)
Average weekly time at fire scene investigations	
Less than 10 h	37 (66.1)
10–19 h	14 (25.0)
20–29 h	5 (8.9)

^†^Differences in sub-total sample due to item non-response or missing. ^‡^Respondent could select all that apply from the response options.

### 3.3 Safety practices, safety training, and exposures

Use of personal protective equipment (PPE) during investigations was reported as often or always by 92.9% (Table 3). PPE types reported included steel-toed boots (89.3%), helmets or hard hats (83.9%), fire-resistant overalls or coveralls (57.1%), and full turnout gear (37.5%). Use of personal protective respiratory equipment (PPRE) during investigations was reported as often or always by 60.7%, sometimes by 32.1%, and never or rarely by 7.1%. Respiratory protection types reported included N95 or P100 respirators (69.6%), SCBA (44.6%), and canister masks (44.6%). Exposure frequency at fire investigation scenes was reported as often or always for soot (89.3%), burned fire debris (73.2%), smoke (67.9%), and hazardous substances (60.7%). Bloodborne pathogen exposure was reported as often or always by 10.9% and sometimes by 34.5%. Preliminary exposure reduction training was reported by 12.5%. PPE cleaning after every incident was reported by 66.1%, and decontamination after every investigation was reported by 53.6%. Decontamination practices specific to fire investigators were reported as present by 43.6% and not present by 56.4%.

**Table 3:** Safety practices, safety training and exposures of participants in the fire investigators’ lived awareness of risk and exposure – a photovoice study on safety and health (*n* = 56).

Characteristics	*n* (%)
Use of PPE during investigation	
Often/Always	52 (92.9)
Sometimes	3 (5.4)
Never/Rarely	1 (1.8)
PPE types worn during investigation^‡^	
Full turnout	21 (37.5)
Fire-resistant overalls/Coveralls	32 (57.1)
Helmet/Hard Hat	47 (83.9)
Steel-toed boots	50 (89.3)
Other PPE	28 (50.0)
Use of PPRE during investigation	
Often/Always	34 (60.7)
Sometimes	18 (32.1)
Never/Rarely	4 (7.1)
PPRE types worn during investigation^‡^	
N95/P100 respirators	39 (69.6)
SCBA	25 (44.6)
Canister mask	25 (44.6)
Cloth mask	9 (16.1)
Other respiratory PPE	5 (8.9)
Frequency exposed to hazard at fire investigation scene	
Smoke	
Often/Always	38 (67.9)
Sometimes	12 (21.4)
Never/Rarely	6 (10.7)
Soot	
Often/Always	50 (89.3)
Sometimes	5 (8.9)
Never/Rarely	1 (1.8)
Hazardous substances (CO, NO2, VOCs, accelerants, asbestos, heavy metals)	
Often/Always	34 (60.7)
Sometimes	18 (32.1)
Never/Rarely	4 (7.1)
Burned fire debris (collapsed buildings/confined space)	
Often/Always	41 (73.2)
Sometimes	13 (23.2)
Never/Rarely	2 (3.6)
Bloodborne pathogens (saliva, blood)	
Often/Always	6 (10.9)
Sometimes	19 (34.5)
Never/Rarely	30 (54.5)
Ever received preliminary exposure reduction training	
Yes	7 (12.5)
No	49 (87.5)
PPE cleaned after each investigation	
Yes, after every incident	37 (66.1)
Yes, but only after certain incidents	19 (33.9)
Mechanism of PPE cleaning^‡^	
Machine washed	44 (78.6)
Wiped down	27 (48.2)
Professionally cleaned	8 (14.3)
Other	8 (14.3)
Frequency of decontamination after fire investigation	
After every investigation	30 (53.6)
After most investigations	19 (33.9)
Occasionally	7 (12.5)
Fire department/agency has decontamination practices for fire investigators	
No	31 (56.4)
Yes	24 (43.6)

^†^Differences in sub-total sample due to item non-response or missing.

PPE = Personal Protective Equipment and PPRE = Personal Protective Respiratory Equipment.

^‡^Respondent could select all that apply from the response options.

### 3.4 Health conditions and health behaviors

Self-rated health was reported as very good (48.2%), good (35.7%), excellent (12.5%), and fair (3.6%) (Table 4). Self-reported health conditions included cardiovascular disease (21.4%), musculoskeletal injuries or disorders (17.9%), mental health conditions (12.5%), diabetes (7.1%), respiratory disease (5.4%), and cancer (5.4%); 42.9% reported none of the listed conditions. Family history was reported for cancer (53.6%) and cardiovascular disease (60.0%). Tobacco use history was reported by 43.6%. Psychosocial factors (i.e., anxiety, depression, stress) were reported as often or always by 28.6% and sometimes by 41.1%. Alcohol consumption frequency was reported as occasional, defined as 1 to 2 times per week (70.0%), never (21.0%), frequently (3 to 4 times per week, 5.4%), and regularly (more than 4 times per week, 3.6%). Mental health resources through the department or agency were reported as available by 89.3%, and seeking professional help for work-related stress or trauma was reported by 34.5%.

**Table 4:** Health conditions and health behaviors of participants in the fire investigators’ lived awareness of risk and exposure – a photovoice study on safety and health (*n* = 56).

Characteristics	n (%)^†^
Self-rated health	
Excellent	7 (12.5)
Very good	27 (48.2)
Good	20 (35.7)
Fair	2 (3.6)
Self-reported health conditions^‡^	
Cardiovascular disease^a^	12 (21.4)
Respiratory disease^b^	3 (5.4)
Cancer	3 (5.4)
Diabetes	4 (7.1)
Musculoskeletal injuries or disorders^c^	10 (17.9)
Mental health conditions^d^	7 (12.5)
Infertility or reproductive health issues^e^	0 (0.0)
None	24 (42.9)
Have a family history of cancer	
No	26 (46.4)
Yes	30 (53.6)
Have a family history of cardiovascular disease	
No	22 (40.0)
Yes	33 (60.0)
Ever used tobacco products	
No	31 (56.4)
Yes	24 (43.6)
Psychosocial factors (anxiety, depression, stress)	
Often/Always	16 (28.6)
Sometimes	23 (41.1)
Never/Rarely	17 (30.4)
Frequency of alcohol consumption	
Never	12 (21.0)
Occasionally (1–2 times/week)	39 (70.0)
Frequently (3–4 times/week)	3 (5.4)
Regularly (>4 times/week)	2 (3.6)
Mental health resources access via department/agency	
No	6 (10.7)
Yes	50 (89.3)
Ever sought professional help for work-related stress/trauma	
No	36 (65.5)
Yes	19 (34.5)

^†^Differences in sub-total sample due to item non-response or missing.

PPE = Personal Protective Equipment and PPRE = Personal Protective Respiratory Equipment.

^‡^Respondent could select all that apply from the response options.

^a^Cardiovascular diseases = stroke, heart attack, hypertension, coronary artery disease, arrhythmia.

^b^Respiratory diseases (asthma, COPD, chronic bronchitis, emphysema, pulmonary fibrosis).

^c^Musculoskeletal injuries or disorders (chronic pain, sprains, osteoarthritis, herniated disc, carpal tunnel syndrome).

^d^Mental health conditions (PTSD, depression, anxiety).

^e^Infertility or reproductive health issues (low sperm count, polycystic ovary syndrome (PCOS), endometriosis).

## 4 Discussion

In this cross-sectional survey of U. S. fire investigators, participants described frequent contact with core post-fire hazards and uneven implementation of exposure controls. Most investigators reported routine use of general PPE during scene work, yet consistent respiratory protection was less universal, even though many described regular exposure to smoke, soot, burned debris, and other hazardous substances. Many participants also indicated that investigator-specific exposure reduction training was uncommon and that agency-level decontamination practices tailored to fire investigation were not consistently in place. Investigators further reported psychosocial stressors as a recurring feature of scene work, and a meaningful subset reported chronic health conditions such as cardiovascular and musculoskeletal disorders, with overweight and obesity being common in this cohort. Taken together, these descriptive findings suggest that investigators may experience recurrent opportunities for both acute exposure and cumulative risk, while organizational support for exposure reduction may lag behind the hazard profile of the work.

The pattern of frequent hazard contact coupled with incomplete respiratory protection is consistent with an expanding evidence base showing that post-fire investigative environments can remain contaminated well beyond active suppression (4, 6). Importantly, these structural and scene-safety hazards are not only theoretical; incident investigations have documented serious injuries and fatalities during fire investigation and investigation-phase operations, including fatal crush injury from chimney collapse and injuries to fire marshal personnel during post-fire structural collapse. In controlled residential fire studies designed to characterize the investigation phase, airborne contaminant levels varied by task and phase, and debris disturbance activities were associated with notable elevations in particulate concentrations, reinforcing that “investigation work” itself can drive exposure through resuspension and off-gassing (4). Field-based evaluations reach similar conclusions in applied settings, including a The National Institute for Occupational Safety and Health (NIOSH) Health Hazard Evaluation that documented formaldehyde and other combustion-related contaminants during fire investigation activities and reported irritation symptoms among some investigators, highlighting plausible short-term respiratory and mucous membrane effects when protection is limited (5). More recent investigator-focused work using silicone wristbands also supports the likelihood of ongoing exposure during routine investigative operations, showing that PAH burden varies with investigation duration and scene recency, which are operationally relevant determinants that may be amenable to targeted controls (6).

Our findings regarding training and decontamination practices are particularly important in light of contemporary contamination-control frameworks for the fire service (20). A hierarchy-of-controls approach emphasizes that effective protection requires layered strategies that extend beyond individual PPE choice to include administrative policies, work practices that reduce contact and resuspension, and systematic decontamination that limits transfer of contaminants to vehicles, stations, and other environments (20). While investigator-specific intervention studies remain limited, the broader fire service literature demonstrates that even when decontamination is attempted, adherence to best-practice steps can be inconsistent (21), suggesting that implementation barriers such as time, resources, role expectations, and organizational culture may materially shape real-world effectiveness (22). In this context, the absence of investigator-tailored decontamination practices reported by many participants should be viewed as an actionable organizational gap, particularly because investigators may transport tools and clothing between scenes and office settings and may re-enter scenes across multiple days, increasing opportunities for secondary contamination.

Dermal exposure is another plausible and under-controlled pathway in fire investigation, given the hands-on nature of scene processing and evidence handling (23, 24). Participants commonly reported frequent contact with soot and fire debris, conditions that support the likelihood of skin contamination and transfer during doffing or equipment handling. Empirical support for this pathway comes from work among firefighters and police forensic investigators at fire scenes showing measurable dermal loading of PAHs using tape stripping methods, with indications that interface areas such as the wrist may be particularly vulnerable (25). These data reinforce that exposure assessment and exposure reduction in investigations, not only inhalation hazards but also glove and sleeve interfaces, hand hygiene, and structured post-scene practices that reduce transfer to skin and personal environments.

Psychosocial hazards also emerged as a salient component of investigator safety, and our survey findings align with growing investigator-specific mental health literature (26). Fire investigation can include repeated exposure to traumatic scenes and high-stakes decision-making under legal and organizational scrutiny, all of which may contribute to stress-related outcomes and influence care-seeking. In a recent cross-sectional survey of U. S. fire investigators, mental health symptoms were common and organizational stigma was strongly associated with PTSD risk, underscoring that worker well-being is shaped not only by individual experience but also by organizational climate and perceived consequences of disclosure (26). In our study, fire investigators reported having access to departmental mental health resources, yet fewer reported seeking professional help for work-related stress or trauma, a pattern that is consistent with stigma and other barriers documented in this workforce and related public safety occupations (27–30).

Finally, the cardiovascular diseases and musculoskeletal disorders reported by investigators in our sample deserve attention as part of a comprehensive occupational health lens. High prevalence of overweight and obesity has been repeatedly documented in firefighter populations and has been linked with clustering of cardiovascular risk factors and duty-related health concerns (31–33), which is relevant given that many investigators transition from suppression roles or maintain operational duties (34). Musculoskeletal injuries and disorders may plausibly reflect cumulative fire service exposures as well as investigative tasks that involve prolonged standing, awkward postures, and repetitive debris manipulation. From a prevention standpoint, these findings support integrating exposure reduction with health promotion, ergonomic considerations, and cardiovascular risk management as part of investigator-specific occupational health programming rather than treating exposures and chronic disease as separate domains. At the same time, interpretation of these chronic health and mental health outcomes warrants caution, as many investigators in this sample reported prior firefighting experience and some reported additional employment, making it difficult to disentangle risks related specifically to fire investigation from cumulative exposures across the broader fire service career course.

This study should be interpreted in the context of several limitations. The sample size was modest given the relatively small size of the fire investigator workforce. Recruitment relied on professional networks and outreach channels, which may not fully capture the diversity of the broader U. S. fire investigator workforce and therefore may limit external generalizability. Geographic context may also have influenced the findings, as investigators working in rural settings, wildland fire environments, or jurisdictions served primarily by volunteer fire departments may be underrepresented in this sample. In addition, no established national surveillance or normative datasets currently exist on the health and safety of U. S. fire investigators, limiting our ability to compare the characteristics of our study sample with those of the broader profession. Further, many participants reported prior firefighting experience and some reported secondary employment, indicating that the self-reported health conditions described in this study may reflect cumulative exposures across multiple occupational roles rather than fire investigator duties alone. Accordingly, these findings should be interpreted as descriptive and exploratory rather than representative of all U. S. fire investigators. Measures were self-reported and cross-sectional, limiting causal inference and introducing the potential for recall or social desirability bias, particularly around sensitive topics such as mental health or PPE practices. At the same time, strengths of the study include its national scope, its focus on an understudied workforce, and its integration within a broader photovoice methodological design that can help contextualize barriers and facilitators to exposure control and health conditions in future qualitative and quantitative studies.

## 5 Conclusion

This brief report provides foundational descriptive evidence that U. S. fire investigators frequently encounter post-fire hazards and that exposure reduction practices, particularly respiratory protection, training, and investigator-specific decontamination policies, may not be uniformly implemented. These observations align with peer-reviewed exposure characterization studies showing that the investigation phase can involve meaningful airborne and dermal contact with combustion byproducts and that task-driven disturbance can shape exposure intensity. Future research should validate the study findings in a larger sample of fire investigators. A prioritization of investigator-specific exposure assessment, evaluation of feasible control strategies tailored to investigative work, and integrated longitudinal surveillance that includes both physical health and mental health outcomes are needed.

## Data Availability

The datasets presented in this study are not publicly available due to privacy and ethical restrictions. Anonymized data, with all participant identifiers removed, may be made available by the authors upon reasonable request and subject to institutional approval.
